# Immune Response in Neurological Pathology: Emerging Role of Central and Peripheral Immune Crosstalk

**DOI:** 10.3389/fimmu.2021.676621

**Published:** 2021-06-10

**Authors:** Austin P. Passaro, Abraham L. Lebos, Yao Yao, Steven L. Stice

**Affiliations:** ^1^ Regenerative Bioscience Center, University of Georgia, Athens, GA, United States; ^2^ Division of Neuroscience, Biomedical Health and Sciences Institute, University of Georgia, Athens, GA, United States; ^3^ Department of Biochemistry and Microbiology, University of Georgia, Athens, GA, United States; ^4^ Department of Animal and Dairy Science, University of Georgia, Athens, GA, United States

**Keywords:** inflammation, neuroinflammation, neurological disorders, neurodegenerative diseases, immune crosstalk, stroke, Alzheimer’s disease, ALS

## Abstract

Neuroinflammation is a key component of neurological disorders and is an important therapeutic target; however, immunotherapies have been largely unsuccessful. In cases where these therapies have succeeded, particularly multiple sclerosis, they have primarily focused on one aspect of the disease and leave room for improvement. More recently, the impact of the peripheral immune system is being recognized, since it has become evident that the central nervous system is not immune-privileged, as once thought. In this review, we highlight key interactions between central and peripheral immune cells in neurological disorders. While traditional approaches have examined these systems separately, the immune responses and processes in neurological disorders consist of substantial crosstalk between cells of the central and peripheral immune systems. Here, we provide an overview of major immune effector cells and the role of the blood-brain barrier in regard to neurological disorders and provide examples of this crosstalk in various disorders, including stroke and traumatic brain injury, multiple sclerosis, neurodegenerative diseases, and brain cancer. Finally, we propose targeting central-peripheral immune interactions as a potential improved therapeutic strategy to overcome failures in clinical translation.

## Introduction

Inflammation is the body’s natural response to injury, infection, or other potential damage, which has evolved as a protective defense mechanism. While acute inflammation is important in helping protect the body from harm, chronic inflammation is often detrimental, and can occur either as an ongoing response to long-term infection or injury or due to amplification of an initial acute response (1). In the central nervous system (CNS), inflammation primarily consists of activation of microglia, the primary immune cells in the brain and spinal cord, in a process commonly referred to as neuroinflammation (2). Neuroinflammation has gained significant attention over the past two decades as a therapeutic target for many neurological and neurodegenerative conditions, such as stroke, traumatic brain injury, Alzheimer’s disease, Parkinson’s disease, and amyotrophic lateral sclerosis (ALS), among others (3).

Specifically, microglia-mediated neuroinflammation has received significant attention as a therapeutic target (4, 5). While microglia and initial immune response has been long thought to be beneficial, they are now commonly thought of as a “double-edged sword,” with dysregulated responses and/or chronic inflammation often preceding and contributing to the onset and progression of many neurological disorders (2, 6, 7). Despite this focus on microglia and neuroinflammation, few therapeutics and clinical trials have been successful in treating these disorders. Early attempts to target microglia to prevent or dampen neuroinflammation typically focused on general immunosuppressants (i.e. minocycline) but were not as effective as in the periphery (8–10). As the role of neuroinflammation continues to be recognized and clinical trials continue to fail (11–13), it is evident that simple treatments aimed at suppressing microglia are not enough, necessitating a more comprehensive approach.

While the CNS has often been considered to be mostly isolated from the peripheral immune system *via* the blood-brain barrier (BBB), a significant role is being recognized for the peripheral immune response in neural pathologies (1, 14). For example, cytokines released in the periphery can both make it across the BBB (15) to cause direct neurotoxicity and contribute to microglia and astrocyte activation (10). Additionally, peripheral immune cells can infiltrate the brain and further affect neuroinflammation, especially after BBB disruption, as seen in ischemic stroke (16). Despite traditional research looking at central and peripheral immune responses as primarily distinct processes, these and other examples contribute to a growing body of evidence for central-peripheral immune crosstalk, especially in pathological conditions (1, 17–20). Perhaps one of the better-known neurological disorders involving the peripheral immune system is multiple sclerosis. Of note, immunotherapies targeting the peripheral immune system have provided some success in treating multiple sclerosis—providing support for this strategy in the aforementioned and other diseases moving forward (21). In this review, we first discuss distinct processes and overlap between the two systems, then provide a comprehensive overview of inflammation (both central and peripheral) in neurological and neurodegenerative conditions, with particular focus on central-peripheral immune crosstalk. Finally, we propose further examination into this crosstalk as a potential avenue for anti-inflammatory therapeutic development.

## The Immune Response in the Central Nervous System: Key Effectors

### Innate Immune System

The central immune system, also known as the neuroimmune system, is comprised of resident macrophages (microglia) and mast cells, as well as other glial cells and neurons (22) (**Table 1**). Microglia, resident macrophages of the CNS, serve as the primary immune cells in the brain and spinal cord. Sharing functions with other macrophages, microglia participate in phagocytosis and undergo activation in response to cytokines and other stimuli. Depending on the stimuli, microglia are activated and exhibit a spectrum of activation states, traditionally divided into M1 (“classically activated”) and M2 (“alternatively activated”) – a designation system used for other macrophages, as well (48). These can generally be thought of as pro-inflammatory and anti-inflammatory phenotypes, respectively, with M1 microglia secreting pro-inflammatory cytokines [e.g., tumor necrosis factor-alpha (TNF-α), IL-6] and M2 microglia secreting anti-inflammatory cytokines (e.g., IL-10). While useful, it is important to note that this distinction is now widely considered an oversimplification, with microglia activation falling on a more complicated axis, including disease-specific activation phenotypes (49, 50). Unlike monocyte-derived macrophages, microglia develop from yolk sac progenitors, migrating into the CNS in early development, and self-renew to maintain their population (36). Microglia exist scattered in a tiled manner throughout the CNS, constantly surveilling the environment for pathogens, damaged cells, and debris. Upon detection, microglia phagocytize the foreign material. In addition to this phagocytic role, microglia are heavily involved in inflammatory signaling *via* extensive cytokine secretion, creating feedback loops to activate nearby microglia and other effector cells (36). Additionally, microglia promote repair after injury *via* anti-inflammatory cytokine secretion and direct communication with neurons at specialized junctions (51). Dysfunction of inflammation-related signaling *via* microglia has been widely associated with chronic inflammation and contribution to many neurological and neurodegenerative conditions (2, 6, 7). It has become increasingly recognized that microglia also contribute substantially to developmental and homeostatic properties, in addition to their immune roles (36).

**Table 1:** Major immune effector cells in neurological disorders.

Cell	Central/Peripheral	Major functions	Associated neurological disorders	References
Microglia	Central	Cytokine secretion, phagocytosis	Most/all	(2, 6, 7)
Astrocytes	Central	BBB maintenance, cytokine secretion, glial scar formation	Most/all	(23, 24)
Mast cells	Both	Allergic reactions, inflammatory signaling, cytokine degradation, gut-brain axis regulation	Alzheimer’s, Parkinson’s	(25–27)
Ependymal cells	Central	BCSFB maintenance, pathogen surveillance, inflammatory signaling	Most/all	(28)
Neurons	Central	Inflammatory signaling *via* cytokines, DAMPs, and neurotransmitters/peptides	Most/all	(29–31)
Neutrophils	Peripheral	Cytokine/protease secretion, BBB breakdown, NET secretion	Most/all	(32, 33)
Basophils	Peripheral	Vasodilation *via* histamine secretion, anti-inflammatory signaling	Most/all	(32, 34)
Monocytes and monocyte-derived macrophages	Peripheral	Cytokine secretion, phagocytosis	Most/all	(35–37)
Natural killer cells	Both	Recognize and attack “non-self” cells, including cancer cells	Glioblastoma, multiple sclerosis	(38–40)
B cells	Peripheral	Immunological memory	Multiple sclerosis	(41)
CD4+ helper T cells	Peripheral	Inflammatory signaling, immunoregulation, neutrophil recruitment	Most/all	(42, 43)
CD8+ cytotoxic T cells	Peripheral	Destroy infected/damaged cells, including cancer cells	Glioblastoma, multiple sclerosis, neurodegenerative diseases	(44, 45)
T regulatory (Treg) cells	Peripheral	Immunomodulation, especially immunosuppression	Most/all	(46)
Gamma-delta (γδ) T cells	Peripheral	Recognize/attack damaged cells, including cancer cells, cytokine secretion	Glioblastoma, stroke, multiple sclerosis	(47)

Astrocytes, primarily known for their wide range of support functions in the CNS, also contribute significantly to the neuroimmune system. Astrocytes, like microglia, undergo activation in response to inflammatory stimuli and interactions with microglia and have been described on an A1/A2 activation spectrum, reflecting microglial M1/M2 activation (23). A1 astrocytes are proinflammatory and neurotoxic and have been implicated in the progression of neurodegenerative diseases and chronic neuroinflammation. A2 astrocytes, however, typically have neuroprotective functions and contribute to protection and repair after insult, similar to M2 microglia (24). In addition to inflammatory and anti-inflammatory roles of reactive astrocytes, astrocytes are vital to the integrity of the blood-brain barrier (BBB; detailed in the next section).

Ependymal cells in the choroid plexus and other ventricular areas are primarily responsible for secreting cerebrospinal fluid (CSF) but have also been noted to have immunological roles, primarily due to their formation of the blood-CSF barrier (BCSFB; detailed in the next section). In addition to this barrier function, several neuroimmune-related functions of ependymal cells and the BCSFB have been realized, including immunological signaling, detoxification, and inflammation/pathogen surveillance (28).

Mast cells are the only hematopoietic immune cells naturally located in the CNS, where they perform similar functions as in the periphery (i.e., allergic reactions and contribution to inflammation) (25). Mast cells have a noted ability to modulate BBB permeability, which often contributes to neuroinflammation and injury after ischemia. However, recently, mast cells have been recognized to degrade proinflammatory cytokines after traumatic brain injury, exhibiting anti-inflammatory and neuroprotective roles (52). These data suggest that mast cells, like many other immune cells, contribute to both inflammatory and anti-inflammatory mechanisms depending on specific conditions, reflecting complex roles and interactions (25). Importantly, mast cells are also the main immune cell involved in gut-brain axis function. Functional gastrointestinal (GI) disorders, including irritable bowel syndrome (IBS) and functional dyspepsia (FD), are now thought to result from gut-brain axis dysregulation (26). As the primary immune cell associated with GI neurons, mast cell activation plays a strong role in GI hypersensitivity and gut-brain inflammation, though more research is needed to determine the extent of mast cell functions and potential as therapeutic targets for these disorders (26). While GI disorders are perhaps the most evident manifestations of gut-brain axis dysregulation, this dysregulation is also increasingly being implicated in neurodegenerative diseases, including Alzheimer’s disease and Parkinson’s disease (27).

Neurons are not always recognized as immune cells, though they themselves interact significantly with immune effector cells, contributing to the neuroimmune response (31). Crosstalk between neurons and glia cells *via* cytokines, for example, can result in nociceptor firing and glial activation (29). As neuronal function is strongly effected by inflammation and the immune responses described above, neurons themselves participate in feedback loops and immunomodulation to coordinate these responses. Neurotransmitter and neuropeptide (e.g., substance P) secretion has also been implicated in regulating cytokine secretion and mast cell activation, suggesting a prominent immunomodulatory role (30).

In addition to native CNS cells, peripheral immune cells play crucial roles in neuroinflammation, especially *via* interactions with CNS cells. Due to their abundance and strong, quick response, neutrophils play an important role in acute infection and injury, as well as immunoregulation of other cells and early stages of tissue repair *via* cytokine and enzyme secretion (32). Neutrophils rapidly infiltrate the CNS upon disruption of the BBB, and while they have some protective effects (e.g., the secretion of neutrophil extracellular traps (NETs) can help trap invading pathogens), matrix metalloproteinase (MMP) and cytokine secretion contributes to further BBB degradation and overall detrimental effects (33). Basophils and peripheral mast cells regulate allergic and inflammatory responses and anaphylaxis *via* histamine secretion – inducing vasodilation to facilitate neutrophil and soluble factor access to injury sites and pathogens (32). More recently, these cells, especially basophils, have been found to secrete significant amounts of interleukin-4 (IL-4), an important mediator of T-helper 2 (Th2) inflammation and anti-inflammatory responses (34).

Monocytes, like granulocytes, are derived from myeloid progenitor cells in bone marrow and participate in phagocytosis and cytokine secretion. After several days in circulation, monocytes migrate into tissues where they differentiate into tissue resident macrophages and myeloid dendritic cells. Tissue resident macrophages go by different names depending on their specific locations (e.g., microglia in the brain) and are found in nearly all tissues and serve important functions in both innate and adaptive immunity. After CNS injury or inflammation, monocyte-derived macrophages can infiltrate the CNS *via* disruption of the BBB, where they can exhibit pro-inflammatory or anti-inflammatory functions depending on the local environment and interactions with microglia determining their activation state (i.e., M1 or M2) (35–37). Specific interactions are detailed in disease sections below.

Myeloid-derived suppressor cells (MDSCs) consist of two populations—monocytic MDSCs (M-MDSCs, monocyte-like) and polymorphonuclear MDSCs (PMN-MDSCs, neutrophil-like)—and have been recognized to play a role in immunoregulation (53). Notably, these cells have many immunosuppressive functions, including T cell suppression, regulatory T cell (Treg) upregulation, and secretion of anti-inflammatory cytokines. The roles of MDSCs in specific diseases, such as MS and cancer, are detailed in later sections.

The last major innate immunity cells are natural killer (NK) cells, not to be confused with natural killer T (NKT) cells, which are derived from lymphoid progenitor cells and function similarly to cytotoxic T cells (detailed in the adaptive immunity section below). More specifically, NK cells have the ability to recognize cells lacking expression of major histocompatibility complex (MHC) class I molecules – found on all “self” cells – allowing them to kill these cells quickly (54). Importantly, they are able to detect cells that cytotoxic T cells and others are not, most notably cancer cells that have lost MHC class I expression (55). In addition, they do not require prior activation to attack these cells, allowing for a more rapid response consistent with other innate immune effector cells. More recently, NK cells have been observed to have adaptive immune functions as well, such as the ability to develop immunological memory (56). Initially thought to be excluded from the brain in healthy conditions, a small CNS-native population has recently been reported (57). Additionally, this population consists mostly of a subset of NK cells with strong cytotoxic functions, though more research is needed to determine the role of this native subpopulation. Given their effector properties, NK cells that infiltrate the CNS in pathological conditions have primarily been associated with cancer (40) and multiple sclerosis (described below) (38, 39), so it is possible that these native cells may also contribute to similar pathologies.

### Adaptive Immune System

The adaptive immune system, unlike the innate immune system, is highly specific, involving recognition and response to specific antigens (32). Due to this specificity and the need for antigen presentation to adaptive immune cells, this response is slower than the innate immune response but is highly effective. Notably, the specificity allows for development of immunological memory, enabling cells to respond to specific pathogens more quickly in the case of future exposure or infection. The primary effector cells of the adaptive immune system include B cells and T cells – also known as B and T lymphocytes – both of which include several important subtypes with various functions (see 41, 46, 58 for more comprehensive reviews of B and T cell subtypes). Importantly, while these cells are typically found in the periphery and involved in the systemic immune response, central-peripheral signaling (e.g., *via* cytokines) and BBB disruption in pathological conditions allows them to infiltrate the CNS, where they can interact with CNS immune cells. As a parallel to microglia activation, T helper (Th) cells, also known as CD4+ cells, assist other immune cells and adopt different profiles after activation, most notably Th1, Th2, and Th17 (42). Th1 cells are generally considered pro-inflammatory, primarily secrete interferon-gamma (IFN-γ), and interact with microglia after infiltrating the CNS. Th2 cells, on the other hand, are generally considered anti-inflammatory and primarily secrete IL-4. While a useful generalization, similar to the M1/M2 microglia model, the Th1/Th2 system has often been viewed as an oversimplification, and newer characterization techniques are being used to describe these populations in more detail (43). A third major subpopulation of Th cells, Th17 cells, develop from a distinct lineage from Th1 and Th2 cells, primarily secrete IL-17, and have recently been recognized as having important immunoregulatory functions, including neutrophil recruitment.

Cytotoxic T cells, also known as CD8+ T cells and killer T cells, primarily recognize and destroy damaged cells, most notably infected cells and cancer cells. CD8+ cells and interactions have crucial roles in glioblastoma and autoimmunity, detailed below. Tregs play an important role in immunosuppression, modulating and ending an immune response to prevent chronic inflammation and autoimmunity. While immunosuppression mechanisms employed by Tregs are under investigation, the immunomodulatory capacity of Tregs has received significant attention for potential therapeutic applications in autoimmune and chronic inflammatory conditions, as well as dysregulated neurological conditions, such as stroke (46).

A final T cell subtype is the gamma delta (γδ) T cell, which carries out both innate and adaptive immune functions (47). Interestingly, T cells mirror many actions of NK cells and can recognize and attack stressed or infected cells, including both solid tumors and hematopoietic cancer cells independent of MHC-binding. Due to this, they have been associated with positive cancer outcomes and received significant attention for cellular cancer therapies (59). While much focus has been on cancer, γδ T cells have also been associated with CNS disorders, primarily stroke and multiple sclerosis (60), where reports on their roles have varied. While most studies have identified proinflammatory roles and detrimental outcomes in these conditions, several others have noted potential reparative effects, as well, indicating that more research is needed on this relatively rare subset (60).

## Blood-Brain and Blood-Cerebrospinal Fluid Barriers

**Figure 1:**
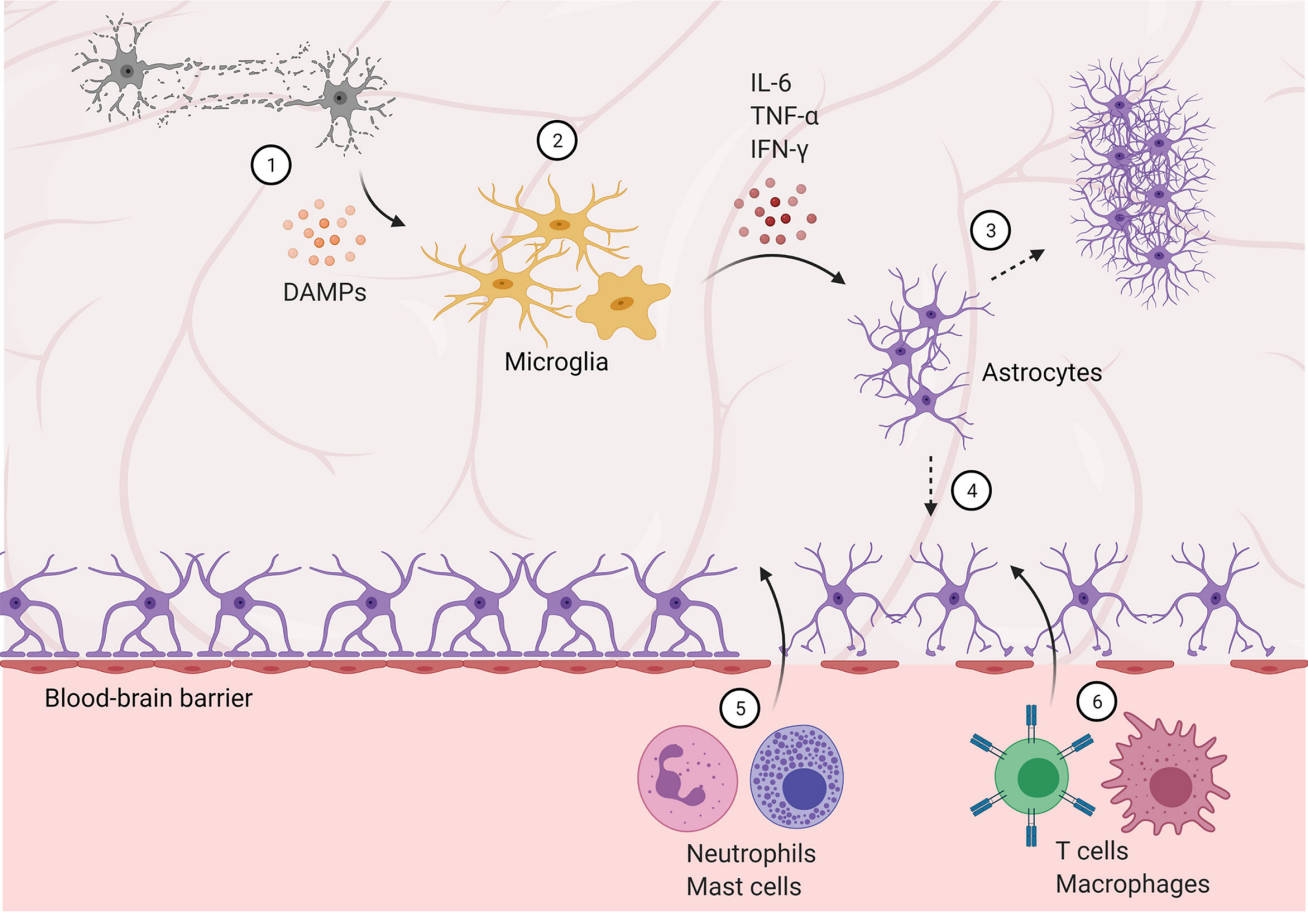
Blood-brain barrier disruption allows peripheral immune cells to infiltrate the central nervous system. (1) Damaged and dying neurons secrete damage-associated molecular patterns (DAMPs), activating microglia. (2) Microglia are polarized to an M1, pro-inflammatory phenotype and secrete pro-inflammatory cytokines and factors, activating astrocytes. (3) Reactive astrocytes form a glial scar, temporarily protecting the brain but preventing future regeneration. (4) Astrocyte activation and dysfunction contributes to blood-brain barrier disruption, allowing infiltration of neutrophils and mast cells in the subacute phase (5), followed by T cells and peripheral macrophages (6) in later stages.

Like the physical barriers (i.e., skin, mucous membranes) of the peripheral immune system, the central immune system has the blood-brain barrier (BBB) and blood-cerebrospinal fluid barrier (BCSFB) as initial lines of defense (61). Necessary neuroimmune maintenance and responses to injury or BBB/BCSFB compromise are carried out by the effector cells described above. These barriers protect the CNS by tightly regulating transport of cells (including peripheral immune cells), small molecules, and ions into the brain (62). Astrocytes are heavily involved in both formation and maintenance of the BBB, and disruption of which is associated with most neurological and neurodegenerative disorders. Maintaining the BBB, therefore, is a vital role for astrocytes in regulating neuroimmune function (**Figure 1**).

Whereas the BBB is formed *via* astrocyte-induced endothelial tight junctions, ependymal cells form tight junctions to create the BCSFB. Similar to the BBB, the BCSFB regulates transport of cells and various molecular components into the brain. As with the BBB, BCSFB disruption is associated with many disorders, and therapeutic strategies to both protect the BCSFB and exploit its transport properties are currently being explored (28).

In acute injury and disorders, such as stroke and traumatic brain injury, integrin expression is decreased, contributing to tight junction degradation and BBB leakage between endothelial cells (63). As astrocytes are activated by pro-inflammatory signaling, they further lose the ability to maintain the BBB, leading to significant BBB breakdown and infiltration of neutrophils and other peripheral immune cells. These cells both further contribute to BBB breakdown, as well as interact with central immune cells and promote secondary injury after stroke.

In addition to acute injuries, it is well understood that disruption of the BBB and BCSFB also occurs in chronic and neurodegenerative diseases, such as ALS, Alzheimer’s disease, and Parkinson’s disease (64). In these diseases, chronic exposure to ROS and dysregulated signaling between astrocytes, CNS endothelial cells, and pericytes cause the barriers to become hyperpermeable, allowing the invasion of monocytes and other peripheral immune cells into the CNS (65, 66). In Parkinson’s disease (PD), increased BBB permeability may be attributed to α-synuclein-induced dysfunction in astrocytes (67) – the PD-associated α-synuclein (A53T) mutation has been found to cause BBB breakdown and neurodegeneration when selectively expressed by astrocytes in a mouse model (68).

General neuroinflammatory processes – both acute and chronic – contribute heavily to BBB breakdown; therefore, the BBB remains a popular therapeutic target (63). After the BBB breaks down, the crosstalk and effects of infiltrating peripheral immune cells are vital to the overall injury response and disease progression in all of these disorders. In the following sections, we describe these specific responses in detail.

## Inflammation in Neurological Disorders

### Stroke and Traumatic Brain Injury

Stroke and traumatic brain injury (TBI) are leading causes of death and disability worldwide, and inflammation is widely recognized as a major contributing factor to secondary injury and pathology post-insult (69–71). While the mechanisms of injury are distinct, both conditions share similar immune responses with potential chronic impact. This long-term pathophysiology can lead to long-term disability and present risk factors for neurodegenerative disease and chronic traumatic encephalopathy (CTE).

As the primary drivers of neuroinflammation in the CNS, microglia have received the most attention for initiation and propagation of this inflammatory cascade; however, astrocytes and neurons have also been implicated, partially as a result of interactions with activated microglia (71–73). Immediately after the initial event, neurons begin to die and release damage-associated molecular patterns (DAMPs), activating microglia, which in turn secrete proinflammatory cytokines which contribute to a feedback loop and initiation of the neuroinflammatory response (69, 70). Astrocytes are induced by this response to become highly reactive, forming a “glial scar” to prevent further damage; however, this glial scar also prevents neuronal regeneration and recovery (23).

In addition to the central immune response, these cytokines and DAMPs are secreted into the circulation, stimulating the peripheral immune system. While the initial peripheral response is temporary, it can lead to immunosuppression and later complications (69). After initial BBB disruption, neutrophils and mast cells both infiltrate the CNS and contribute further to BBB breakdown *via* protease (i.e., matrix metalloproteinase and gelatinase, respectively) secretion. In addition to protease secretion, mast cells secrete cytokines and other vasodilatory and pro-inflammatory factors (e.g., histamine and heparin), further contributing to edema, sustained neuroinflammation, and peripheral immune cell chemoattraction (25, 74). This role is supported by decreased edema and reduced pathology in mast cell-deficient rodent models. Interestingly, however, recent evidence in mast cell-deficient rodents experiencing exacerbated pathology after traumatic brain injury points to anti-inflammatory and neuroprotective roles for these cells, suggesting a more complex role and interactions with other immune cells in the CNS (i.e., microglia and macrophages) (52). After invading the CNS *via* disruption of the BBB, neutrophils generate reactive oxygen species (ROS) that contribute to post-stroke pathology (75). Specifically, ROS and proteinase 3 (PR3) secretion directly modulate microglia, amplifying pro-inflammatory and neurotoxic effects (76). These effects have been strongly correlated to decreased functional outcome following ischemia (77). Microglia have been observed to trap and phagocytize infiltrating neutrophils, alleviating neutrophil-induced neurotoxicity *in vitro* (78). *In vivo*, this microglial association has also been observed, and preventing neutrophil infiltration *via* antibody blockade ameliorated post-stroke behavioral deficits – providing evidence for the functional importance of these neutrophil-microglia interactions (77). An alternative strategy targeting this neutrophil-microglia feedback loop has been explored in pre-clinical studies, utilizing immunomodulation to convert neutrophils to an “N2” phenotype, mirroring the anti-inflammatory M2 microglial phenotype (79). Targeting neutrophils with a peroxisome proliferator-activated receptor-γ (PPARγ) agonist, rosiglitazone – which has previously shown neuroprotective capacity in stroke models (80–82), as well as M2-polarizing effects (83–85) – induced an N2 phenotype resulting in neuroprotection. Interestingly, rosiglitazone treatment increased neutrophil infiltration, and rosiglitazone-treated neutrophil depletion abrogated neuroprotective effects, highlighting the specific neuroprotective effect of N2 neutrophils and demonstrating a novel strategy to modulate traditional detrimental neutrophil-microglia crosstalk.

In addition to neutrophils, T cells (86, 87) and NK cells (88) have also been implicated in post-insult damage, though interestingly, the T cells do not require antigen recognition for these effects. Indeed, blocking T cell invasion *via* antibody blockade demonstrated a decrease in lesion volume, suggesting a prominent role in post-stroke pathology (77). Of note, while T cell blockade appeared to correlate strongly to lesion size, neutrophil blockade was associated more strongly with improved behavioral outcomes, providing evidence for distinct roles and interactions between these cells and native parenchymal cells (77). γδ T cells, despite residing primarily in the gut, have also been demonstrated to play a strong role in post-stroke pathology (89). In concert with Th17 cells, γδ T cells secrete IL-17 and other pro-inflammatory cytokines and have been observed to migrate to meningeal compartments in response to ischemia (90). In the brain, γδ T cell-secreted IL-17 acts with microglia-secreted TNF-α to induce astrocytic expression of the neutrophil chemoattractant CXCL1, contributing to the neutrophil-induced pathology described above (87). Reducing γδ T cell activity *via* fecal transplant (90) or anti-IL-17 blockade (87) ameliorated ischemic injury in mouse middle cerebral artery occlusion (MCAO) models.

Alternatively, T cells and infiltrating macrophages have been noted to promote neurogenesis and encourage post-stroke recovery and repair, suggesting more complex interactions with neural cells (91). More specifically, Tregs, γδ T cells, and M2 macrophages have been shown to enhance neuroprotection *via* reduction of neuroinflammation after stroke (91–93). This is in accordance with evidence from a study investigating extracellular vesicles as a potential stroke therapeutic, in which improved recovery and survival were correlated with increased Tregs and M2 macrophages, and decreased pro-inflammatory Th17 cells (94). Furthermore, several studies have suggested that microglia–T cell interactions may be responsible for determining beneficial or detrimental effects of T cells post-injury (17). Particularly, M1 microglia induce Th1 and Th17 T cell activation (95) and recruit T cells *via* chemokine secretion, creating a pro-inflammatory response and feedback loop (96); alternatively, M2 microglia induce Th2 and Treg activation (95–97) to promote an anti-inflammatory, reparative response. These complex interactions in both responses are regulated by a host of contact-mediated (e.g., MHCII, CD40) and non-contact-mediated factors (e.g., cytokines, chemokines) to balance proinflammatory and anti-inflammatory activity (17).

**Figure 2:**
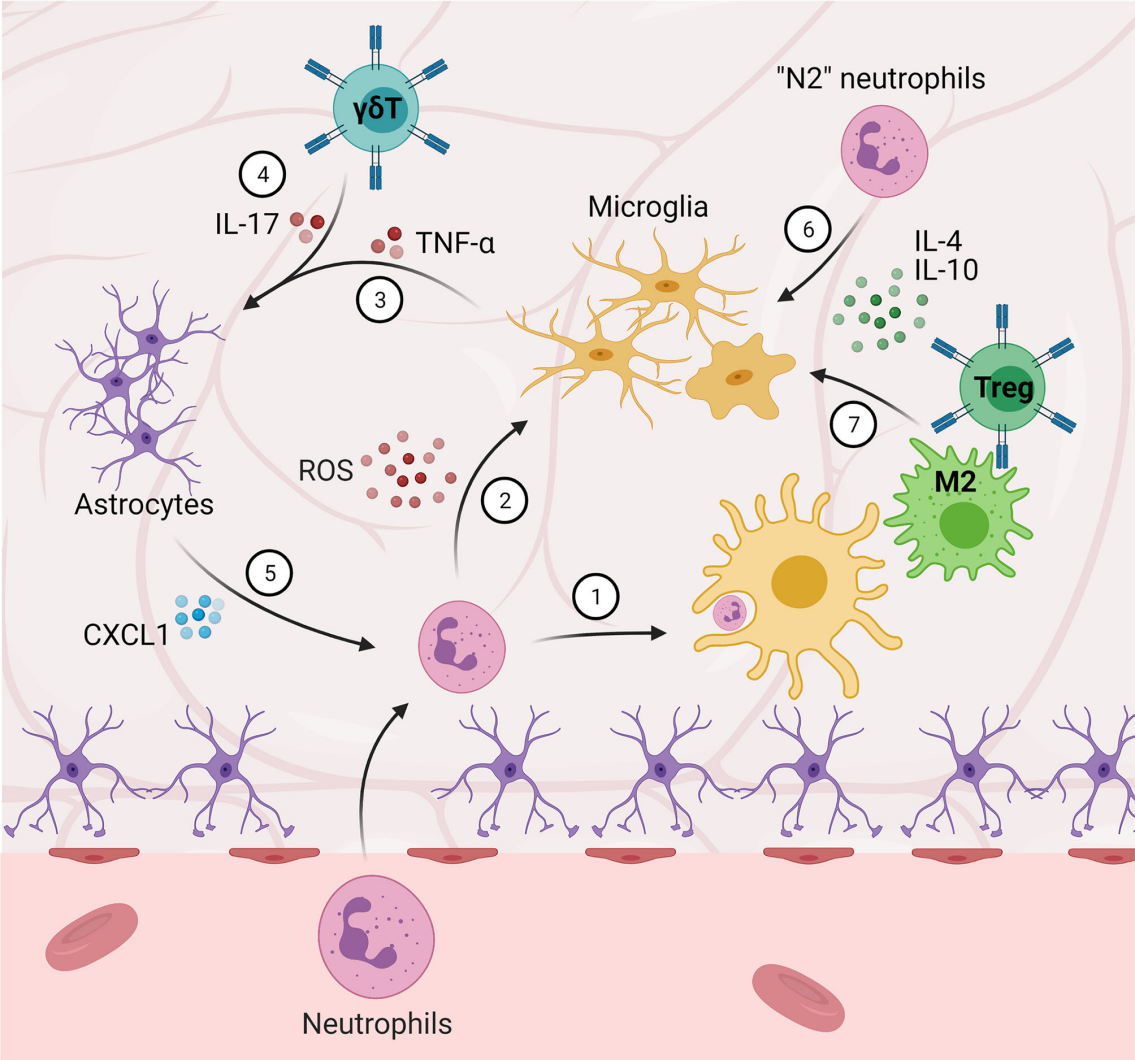
Central-peripheral immune crosstalk in stroke and traumatic brain injury. (1) Microglia phagocytize infiltrating neutrophils. (2) Neutrophils that are not phagocytized secrete ROS and proteinases, amplifying M1 microglial activation. (3-5) Microglia-secreted TNF-α and γδ T cell-secreted IL-17 induce astrocytic expression of CXCL1, which further contributes to neutrophil-induced pathology. (6) “N2” neutrophils, as well as (7) M2 macrophages and Tregs, promote M2 microglial activation and anti-inflammation.

Despite the focus on inflammation and immunomodulation and promising pre-clinical results, translation has been largely unsuccessful (89, 98). Due to the mounting evidence of the importance of peripheral–central immune crosstalk (**Figure 2**), targeting the peripheral immune system in addition to the central immune system, particularly the types of interactions outlined here, represents significant potential for improving translational potential (99).

### Multiple Sclerosis and Autoimmune Disorders

Autoimmune disorders, such as multiple sclerosis (MS), involve a direct attack by the immune system on host cells. In MS, immune cells attack the myelin sheath surrounding axons, ultimately leading to demyelination and disability. MS has traditionally been distinguished into two categories – relapse-remitting or progressive; however, it is now recognized that these categories overlap, creating a spectrum of disease manifestation (100). Relapsing symptoms can be attributed to acute inflammation, especially involving peripheral immune infiltration, while progressive symptoms are mostly attributed to neuroinflammation. It is worth acknowledging that there has been moderate success in treating MS with immunotherapy, especially compared to other neurological diseases; however, these therapies primarily target the peripheral immune system and thus are not very effective at treating progressive symptoms (21). The recognition of overlap between these symptoms indicates a crucial role of central and peripheral immune crosstalk in MS onset and progression and may serve as an improved therapeutic target for progressive MS. While many mechanisms of MS etiology remain unknown, experimental autoimmune encephalomyelitis (EAE) models have been widely used to study MS and have revealed potential mechanisms. One common mechanism initially commences with CD4+ T cell migration into the CNS (101). This infiltration can occur due to BBB disruption or across the BCSFB, and these T cells can either be autoreactive – recognizing host myelin as an antigen – at the time of infiltration or subsequently activated. In either case, these reactive T cells secrete proinflammatory cytokines, stimulating microglia and recruiting peripheral macrophages that contribute to myelin degradation (101). Activated microglia secrete additional proinflammatory cytokines, worsening neuroinflammation and further disrupting the BBB, allowing increased peripheral immune cell infiltration. This neuroinflammation is mediated *via* both microglia and astrocytes (102) and is maintained and perpetuated throughout disease progression as neurons demyelinate and die off (103).

Despite a focus on T cells as the primary autoimmune effector cells in MS, recent evidence strongly implicates B cells, as well (104). Upon inflammation, B cells can infiltrate CNS compartments, and increased CSF B cell counts are correlated with MS lesions. Additionally, B cells are associated in higher numbers with early demyelinating lesions rather than later disease stages, suggesting a role in disease progression. As mentioned above, immunotherapies have shown success for MS, notably *via* specific targeting of B cells (105). While these results are promising, much work remains concerning B cell-targeted therapies and mechanisms underlying the role of B cells in MS (104); however, recent evidence points to a mechanism by which B cells contribute to T cell autoreactivity and proliferation, thus facilitating MS onset and progression (106). This study further supports the potential to develop B cell-targeting approaches. Along these lines, it has now been discovered that MDSCs may regulate B cell activity in the CNS (107). Specifically, lower PMN-MDSC counts in the CSF were associated with MS relapse compared to higher counts in stable conditions. MDSC depletion in an EAE model prevented recovery, providing evidence of the immunosuppressive capacity of MDSCs in MS. Finally, it was shown that MDSCs suppress B cell activation and accumulation, ultimately resulting in decreased microglial activation and disease severity (107).

MS patients have a noted component of inflammation in the meningeal compartments that persists throughout all stages of disease progression (100). The extent of meningeal inflammation is correlated to disease severity and is primarily composed of CD4+ and CD8+ T cells, B cells, and dendritic cells (108). These aggregates are maintained *via* proinflammatory cytokines and signals generated both by immune cells in the meningeal compartment, as well as in the CNS, including astrocytes in MS lesion areas (109). Heightened meningeal inflammation has also been correlated to increased microglial activation and neuroinflammation, resulting in downstream demyelination and neuronal death (110). Crosstalk between this meningeal compartment and neuroimmune cells, therefore, represents a potential target to treat progressive MS. Indeed, several studies targeting neuroinflammation – especially reactive astrocytes – appear to alleviate progressive MS in preclinical models (111–113). Additionally, a few of the FDA-approved immunotherapies for relapsing MS that have shown some benefit in progressive cases have primarily been successful when patients were in early stages of progressive MS (114). This suggests that interrupting or preventing the eventual inflammatory meningeal compartmentalization and neuroinflammation could effectively delay or prevent progressive symptoms (21).

While pro-inflammatory microglia activation and responses are associated with detrimental outcomes, a recent study using CX3CR1-Cre to specifically label and distinguish microglia from macrophages has provided evidence of a previously unrecognized protective role of microglia (115). Particularly, microglia appear to limit peripheral macrophage infiltration into the CNS, protecting from neurotoxic effects and demyelination. This effect was eliminated *via* microglia ablation, resulting in increased macrophage invasion and axonal loss. Additionally, microglia in this model were determined to exhibit unique activation phenotypes, more consistent with “disease-associated microglia” (DAMs) previously associated with Alzheimer’s disease (116). This indicates that these microglia-macrophage interactions may be similar to those seen in other neurodegenerative diseases. This crosstalk, therefore, plays an important role in both onset and long-term progression of MS and may serve useful for therapeutic intervention.

Finally, neutrophils have also been implicated in MS progression and severity. High neutrophil counts and neutrophil-to-lymphocyte ratio has been directly correlated to disease activity in MS patients (117). Supporting this, neutrophil depletion in EAE models of MS successfully reduced disease severity—lending further credence to a detrimental role of neutrophils in MS and other autoimmune disorders (118). Probing into this mechanism, CXCR2-knockout EAE mice were also found to exhibit reduced disease severity, suggesting neutrophil-induced damage may be mediated *via* CXCR2 signaling and interactions with microglia and providing a potential therapeutic target (119).

## Inflammation in Neurodegenerative Diseases

### Amyotrophic Lateral Sclerosis (ALS)

Amyotrophic lateral sclerosis (ALS), commonly referred to as Lou Gehrig’s disease, is a progressive neurodegenerative disease characterized by the degeneration of motor neurons in the motor cortex, brainstem, and spinal cord. Motor neuron dysfunction in ALS patients results in severe impairment of motor functions including mobility, speech, respiration, and the ability to eat. These symptoms cause those suffering from the condition to experience a low quality of life and an average life expectancy of only two to four years following onset (120). Greater than 90% of ALS cases arise sporadically, however, familial cases have been linked to several genetic mutations, most notably mutant Cu2+/Zn2+ superoxide dismutase (mSOD1), which also appears in idiopathic cases (121). The pathogenesis of ALS is heterogeneous, implicating and causing damage to a wide variety of systems, pathways, and cell types. This heterogeneity has presented challenges for developing treatments – current therapeutics do not successfully address multiple pathologies simultaneously, are few in number, and do not significantly improve length or quality of life (122). Thus, further elucidation of complex disease mechanisms and the cell types involved is vital to creating targeted and multi-faceted treatments for ALS.

Microglia are the principal contributors to neuroinflammation and are neuroprotective in early stages of ALS (123). However, as the disease progresses, microglia become prone to multiple stressors, such as oxidative stress and mitochondrial dysfunction. These stressors culminate to activate microglia, causing them to adopt a pro-inflammatory phenotype, resulting in secretion of pro-inflammatory cytokines (i.e., IL-6, IL-1β, TNF-α) that contribute to motor neuron death (124, 125). This response is regulated by the activation of nuclear factor kappa-light-chain-enhancer of activated B cells (NF-κB) pathway (126). Thus, microglia play a critical and dual role in ALS, and their potential to either combat or encourage neurodegeneration has made them significant targets for developing therapeutics (127).

Astrocytes are also highly implicated in the neuroinflammatory response of ALS and become activated as a result of free radicals and M1 microglial signaling *via* pro-inflammatory cytokines (128, 129). mSOD1 astrocytes have been found to activate NOX2, causing superoxide production leading to neuronal loss (130). Additionally, transplanted healthy astrocytes have been shown to reduce microgliosis in an ALS mSOD1 mouse model, indicating that astrocytes may regulate the inflammatory response of microglia and their downstream effects on motor neuron survival (131).

As mentioned above, BBB disruption also allows for peripheral immune infiltration. Degeneration at the neuromuscular junction (NMJ) results in recruitment and infiltration of peripheral macrophages along nerve fibers – autopsies of patients with ALS show infiltrating neutrophils and degranulating mast cells in the NMJ and along the entire peripheral motor pathway (132, 133). Mast cells were identified both in close association with NMJs and formed extracellular traps *via* interactions with neutrophils contributing to neurodegeneration in ALS subjects (133). This mechanism was supported by evidence that blocking mast cell and neutrophil migration to the NMJ resulted in decreased degeneration and muscle loss. However, other evidence shows this to be a response to denervation rather than an initiator – similar to the DAMP-induced response observed in stroke and TBI (134). Circulating monocytes display a proinflammatory phenotype in ALS patients and higher proinflammatory gene expression (e.g., IL1B, IL8, FOSB, CXCL1, CXCL2) is correlated with more rapid disease progression (135). Notably, these monocytes were observed to be preferentially recruited to the spinal cord than the brain in the mouse SOD1 model, in accordance with the loss of lower motor neurons in the spinal cord but not upper motor neurons in the brain. This inflammatory monocyte activation was also observed 2 months prior to disease onset, suggesting an important role for monocyte activation and infiltration in disease onset and progression upon recruitment by resident microglia. Several lines of evidence from human patients support this role of monocyte activation and interactions with microglia, as well – particularly, increased expression of CCL2 (a monocyte-recruiting chemokine) was detected in ALS patient glia, and CD14+ monocytes were detected in close proximity to motor neurons and correlated with worsened disease progression (136). A concomitant decrease in circulating CD14+ monocytes was noted in ALS patients, hypothesized to correspond to these cells migrating to the brain and spinal cord (137, 138).

In addition to the innate peripheral response, the adaptive immune response is involved *via* infiltrating and circulating T cells in ALS (20). In early disease stages, Tregs appear to be more involved, playing a prominent immunosuppressive role (139, 140). In later stages, however, proinflammatory Th1 and CD8+ T cells have been found to be upregulated and contribute to cytotoxicity *via* release of IFN-γ and TNF-α (44, 45). Accumulating evidence shows that significant crosstalk takes place between the central and peripheral immune systems during ALS pathogenesis (140, 141). As mentioned above, Tregs can suppress microglial activation *via* IL-4 secretion, however as the disease progresses, neuroprotective Tregs and M2 microglia shift to a proinflammatory Th1/M1 response (142). Th1 cells secrete IFN-γ which further promotes M1 microglia activation, and M1 microglia in turn promote Th1 cell activation, creating a positive neuroinflammatory feedback loop (143). This process is strongly associated with decreased FOXP3 expression by Tregs (144). Additionally, astrocytes in ALS over-produce transforming growth factor-β1 (TGF-β1), suppressing the neuroprotective response of infiltrating T-cells and accelerating disease progression (145).

Though there are conflicting arguments for whether the brain-gut biota axis contributes to ALS pathogenesis, alterations in gut biota have been shown to precede symptoms of motor dysfunction and muscle atrophy in mSOD1 mice (146). In response to growing evidence for crosstalk between the gut microbiota, neuro, and peripheral immune systems, a clinical trial is investigating fecal microbial transplantation (FMT) as a potential treatment for patients with ALS. The results have not yet been published, but it is hypothesized FMT may increase the number of Tregs and subsequently result in downstream neuroprotective effects on motor neurons (147).

**Figure 3:**
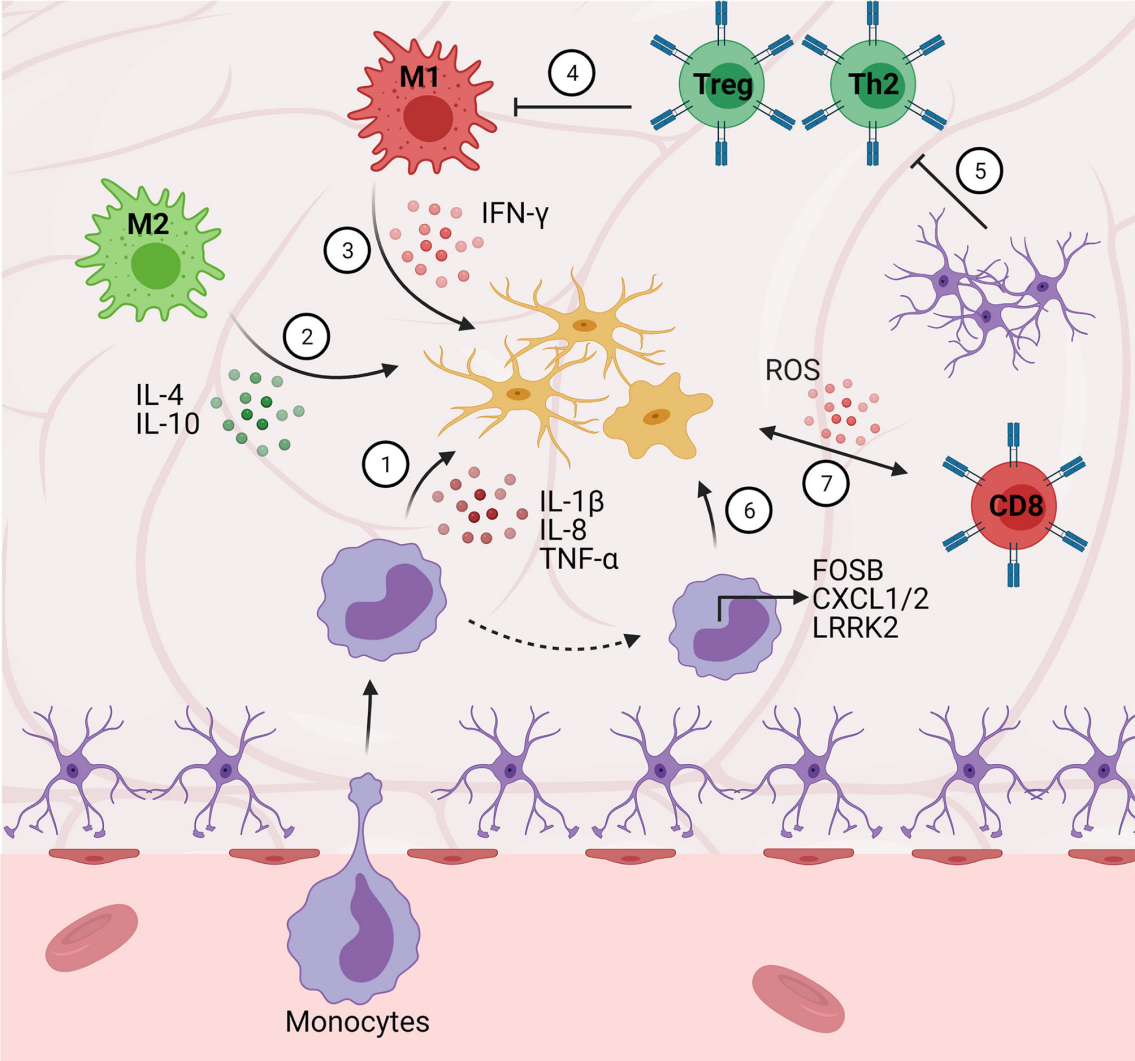
Central-peripheral immune crosstalk in neurodegenerative diseases. (1) Infiltrating monocytes secrete pro-inflammatory factors that activate microglia (e.g., IL-1B, IL-8, FOSB, CXCL1/2). (2) M2 macrophages play an early neuroprotective role *via* anti-inflammatory cytokine (e.g., IL-4, IL-10) secretion. (3) Alternatively, M1 macrophages play a late neurotoxic role *via* secretion of pro-inflammatory cytokines (i.e., IFN-γ). (4) Peripheral Th2 cells downregulate pro-inflammatory cytokine secretion (e.g., GM-CSF, TNF-α, IL-2) resulting in decreased microgliosis. (5) Astrocytes secrete TGF-β1, suppressing neuroprotective T cell responses (ultimately disinhibiting M1 pro-inflammatory responses). (6) Monocytes upregulate chemotactic gene expression (e.g., LRRK2), contributing to pro-inflammatory pathology. (7) Cytotoxic (CD8+) T cells stimulate microglial ROS secretion.

It is apparent that crosstalk between systems plays a crucial role in the onset and development of ALS and other neurodegenerative diseases (**Figure 3**). Further research into developing therapeutics should target interactions between T-lymphocytes, microglia, astrocytes, and the gut microbiota in the neuroinflammatory response.

### Alzheimer’s Disease

Alzheimer’s Disease (AD) is the most prevalent neurodegenerative disorder and the leading cause of dementia (148). AD pathogenesis is characterized by the accumulation of extracellular amyloid-beta (Aβ) plaques and neurofibrillary tangles (NFT) in cortical and limbic brain regions (149). Aβ plaques and NFT act as danger-associated molecular patterns (DAMPs), triggering a neuroinflammatory response from resident CNS immune cells, including microglia (150, 151). This response may be initially protective, as activated microglia have been shown to clear excess Aβ plaques and cell debris (152, 153). However, akin to other neurodegenerative diseases, AD microglia become chronically overactive and the continued release of pro-inflammatory cytokines leads to neurotoxicity, synapse loss, cognitive decline, and dysfunction in the microglia themselves (154). Notably, evidence shows that phagocytic clearance of Aβ plaques by microglia becomes impaired in AD as a result of high cytokine concentrations (155). Thus, Aβ plaque clearance and combatting neuroinflammation *via* microglia are key targets in existing and developing therapeutics.

Astrocytes undergo astrogliosis as a result of microglial-mediated NF-κB signaling, becoming active and further contributing to neuroinflammation (23, 156). Astrocytes can secrete Aβ, and in turn, Aβ plaques have been shown to stimulate astrocytic release of AD-associated proinflammatory cytokines (IFN-γ, IL-1β, TNF-α, IL-6, and TGF-β). Along with microglia and oligodendrocyte precursor cells, astrocytes contribute to the formation of glial scars, as seen in stroke and TBI. In AD patients, glial scars can initially be neuroprotective by helping to phagocytose Aβ plaques and constrain toxic materials resulting from NFT-damage (157, 158). However, the scars reduce neural plasticity and can serve as a barrier to neurogenesis and replacing lost neurons, hindering recovery (159). Modulating the dual role of glial scar formation therefore has the potential to mitigate the effects of AD.

Mast cells are also hypothesized to have pro-inflammatory and detrimental roles in AD, both directly and *via* interactions with microglia (160). The NLPR3 inflammasome is activated downstream of NF-κB and can promote chronic inflammation. Due to this, the NLPR3 inflammasome has received attention as both a biomarker and target to treat early-stage AD, primarily in microglia. Mast cells have also been shown to express the NLPR3 inflammasome in other models (161) and are postulated to contribute to AD-associated inflammation *via* similar mechanisms, positioning them as a potential target for therapeutic intervention (160).

Systemic and infiltrating peripheral immune cells have also been implicated in AD pathogenesis, both encouraging and abating disease progression directly (e.g., phagocytosis) and by affecting neuroinflammation *via* crosstalk with the CNS. Aβ-reactive B cells and T cells have been found in peripheral blood of patients with AD, suggesting that Aβ can antigenically induce an adaptive immune response (162, 163). Additionally, evidence has shown levels of circulating Aβ-reactive T cells to be positively correlated with AD progression (164). Immunosuppressive Tregs have been found to be decreased in blood samples from AD patients, indicating their loss may contribute to the failure in regulating inflammation (165). Furthermore, Aβ specific Th2 cells in the periphery can downregulate pro-inflammatory cytokines (GM-CSF, TNFα, IL-2) in transgenic mice – a shift that is correlated with reduced microgliosis and improved cognitive performance (166). Notably, peripheral macrophages have also been increasingly implicated in Aβ clearance, and CNS-expressed IL-34 appears to reduce this clearance capacity (167). Further examination of interactions between resident CNS cells and infiltrating macrophages – and the differences in Aβ clearance ability – may serve as an effective target for therapeutic intervention.

The increased permeability of the BBB in AD allows for infiltration of T lymphocytes at postcapillary vessels, adding to the neuroinflammatory milieu. VCAM-1, RAGE receptors, and CCR5 chemokine receptors mediate the recruitment of T cells *via* Aβ stimulation from the brain parenchyma (168. T-cell migration across the endothelium is additionally promoted *via* Aβ-induced TNF-α secretion by microglia (169). This results in astrocytic activation and overproduction of TGF-β1, potentially as a compensatory mechanism to decrease Aβ accumulation, though this response is ultimately detrimental (170). Treatments may consider targeting crosstalk between T cells, microglia, astrocytes, and Aβ to attenuate peripheral recruitment to the BBB and exacerbation of neuroinflammation in AD.

The choroid plexus comprises the blood-CSF barrier and is a master regulator of bidirectional communication and immune trafficking into the CNS (171). In AD models, the choroid plexus appears to be immunologically suppressed due to insufficient IFN-γ signaling (172). Utilizing Tregs to target this immunosuppression in the 5xFAD model leads to increased trafficking of monocytes and improved function (173). Dysfunction in the choroid plexus therefore does not allow sufficient entry of beneficial leukocytes, while neutrophils are still able to pass through the compromised BBB and further potentiate disease (174). Future studies should continue investigating the therapeutic potential of the choroid plexus as a significant point of crosstalk between the central and peripheral immune systems in AD.

### Parkinson’s Disease

Second in prevalence for neurodegenerative disorders is Parkinson’s Disease (PD), of which the incidence is expected to double by 2040, primarily due to an aging worldwide population (175, 176). PD is characterized by the degeneration of nigrostriatal dopaminergic neurons, leading to symptoms of bradykinesia, resting tremor, impaired balance, and rigidity (177). Patients also may experience non-motor disturbances (hyposmia, constipation, REM sleep disorders) in a prodromal phase preceding motor dysfunction by many years – this stage is intimately linked to peripheral inflammation and has become a time point of interest for treating PD prior to irreversible damage (178). The hallmark feature of PD is the pathogenic accumulation of Lewy bodies, which are composed of aggregated and misfolded α-synuclein protein. PD α-synuclein is liable to aggregate due to its β-sheet-rich amyloid-like structure and conformational changes induced by C-terminal truncation, serine 129 phosphorylation, and ubiquitination (179). Familial cases of PD have been causally linked to point mutations in the α-synuclein gene (SNCA), though the majority of cases arise sporadically (180, 181). α-synucleinopathy is nevertheless seen both in inherited and idiopathic cases, being a key contributor to mitochondrial dysfunction and activator of proinflammatory responses (182).

Following the pattern of other neurodegenerative diseases, microglia in PD become overactive and contribute to a chronic, deleterious cycle of neuroinflammation *via* secretion of TNFα, IL-6, IL-1β, IFN-γ and free radicals (ROS, NO) which stimulate NF-κB cell-death pathways (183). It is well understood that α-synuclein can directly activate microglia and trigger pro-inflammatory behavior – α-synuclein provokes dose-dependent activation of microglia in primary cultures (184). A longitudinal study of idiopathic PD patients found no significant changes in microglial activation, and activation of microglia has been shown to precede dopaminergic degeneration and motor dysfunction in transgenic mice (184, 185). Thus, robust evidence exists for microglia-mediated neuroinflammation having a primary role in PD pathogenesis, making it a key target for past and emerging therapeutics.

Postmortem PD brains display an elevated density and phenotypic changes in astrocytes (186), which directly communicate with microglia and exacerbate their inflammatory behavior when activated (187). Astrocytes can endocytose and degrade neuronally secreted α-synuclein (188, 189), however, studies show that high concentrations of α-synuclein dose-dependently induce a proinflammatory response from astrocytes (190). Therefore, the ability of astrocytes to remove α-synuclein in PD may become impaired at high extracellular concentrations of the aggregate, resulting in the formation of α-synuclein inclusions in astrocytes and their activation (among other aberrations) which significantly contributes to pathology (191, 192).

As previously mentioned, the peripheral immune system is thought to be highly implicated in the development of preclinical nonmotor symptoms in PD –serum TNF-α levels were shown to be significantly correlated with prodromal symptom severity in PD patients (193). TNF-α and TNF-α receptor 1 serum levels are also elevated in patients with PD compared to healthy individuals (194, 195). The expression of genes associated with immunoregulation and leukocyte migration, notably LRRK2, are upregulated by peripheral monocytes in PD and may be correlated to disease severity in early stages (19, 196). Furthermore, α-synuclein deposits have been found in several peripheral tissues such as the cardiovascular system, GI tract, skin, and retina (197). Therefore, it is clear that systemic inflammation plays an integral part in PD etiology, even prior to BBB/BCSF disruption and particularly in the disease’s initial phase.

Consistent with other neurodegenerative disorders, reduced BBB integrity facilitates the entry of peripheral monocytes and lymphocytes into the CNS. Studies using post-mortem PD mouse brains have shown evidence of neurodegeneration resulting from infiltrating CD4+ and CD8+ T cells. Both CD4+ T cells and B lymphocytes are decreased in peripheral PD blood, however, B lymphocytes do not appear to invade the CNS or be significantly associated with pathogenesis (198–200). Chronic T cell infiltration can exacerbate microglial activation (201). Additionally, microglia may become “primed” in PD and aged brains, displaying high expression of MHC class II receptors and responding more aggressively to peripheral immune stimulation than healthy controls (202, 203). Transfer of activated Tregs provides neuroprotection in PD animal models, while T effector cells have been found to stimulate ROS secretion by microglia (204).

Finally, it is well established that dysregulation in the gut-brain axis contributes to PD onset and progression (27). Gut inflammation is common in patients who develop PD, and most individuals experience GI disturbances before motor symptoms appear (205, 206). The Braak theory proposes that PD may initiate in the GI tract and advance to the brain *via* the vagus nerve. Though this hypothesis is currently under investigation, an epidemiological study has found a decreased risk of developing PD to be associated with full truncal vagotomies (207). Therefore, when looking at the role of crosstalk in PD pathogenesis, future investigations must consider interplay between the central immune, peripheral immune, and gut-brain axis to develop comprehensive, immunomodulatory interventions.

## Inflammation in Brain Cancer

### Glioblastoma

**Figure 4:**
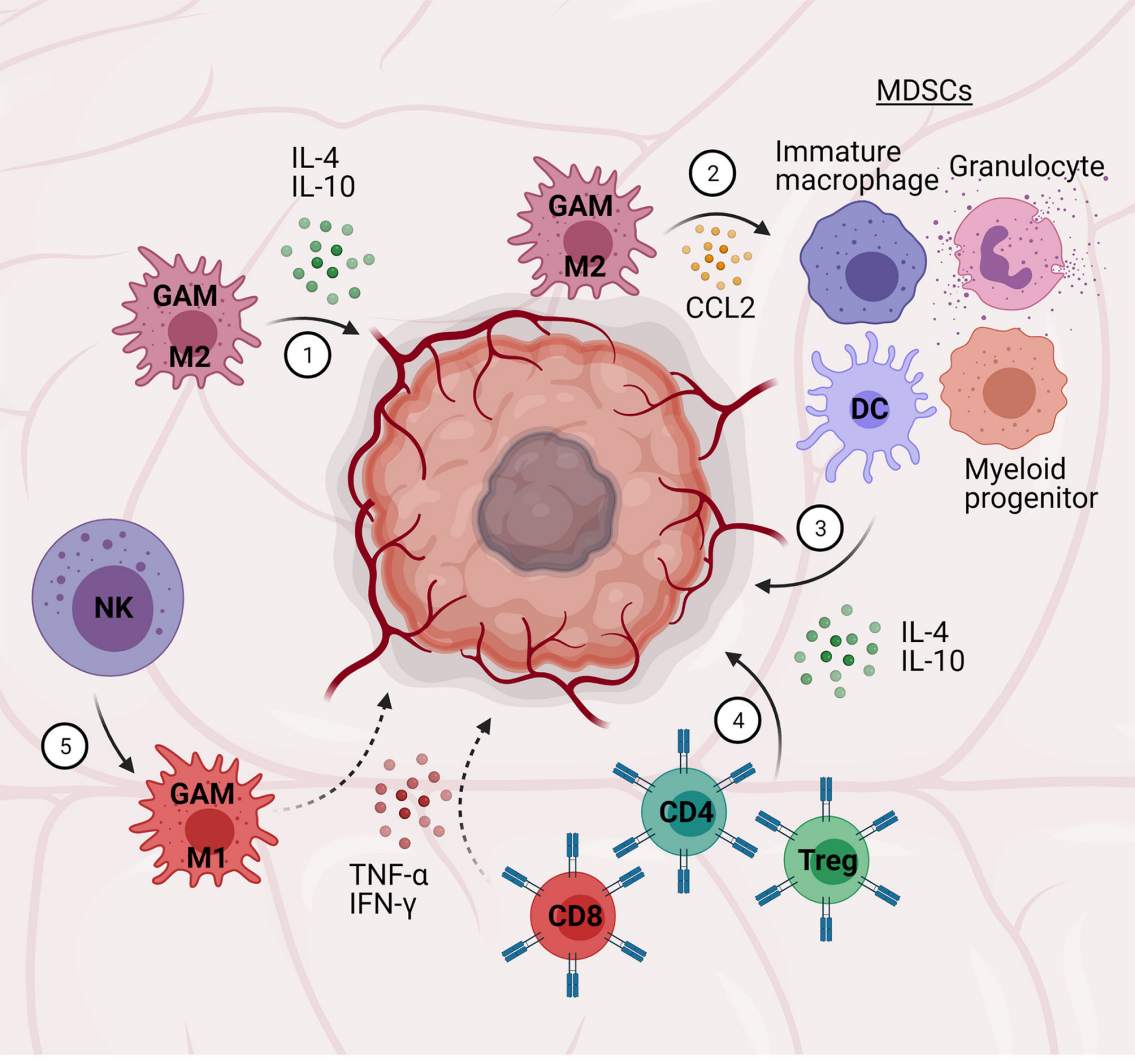
Central-peripheral immune crosstalk in glioblastoma. (1) Glioma-associated microglia/macrophages (GAMs) adopt M2 phenotypes and secrete pro-tumor, anti-inflammatory factors (i.e., IL-4, IL-10). (2) GAMs secrete chemokines (CCL2) to attract myeloid-derived suppressor cells (MDSCs; immature macrophages, granulocytes, dendritic cells, myeloid progenitors). (3) MDSCs secrete pro-tumor, anti-inflammatory cytokines (e., TGF-β, IL-10). (4) CD4+ T cells and Tregs further contribute to a pro-tumor, anti-inflammatory microenvironment, while CD8+ T cells contribute to an anti-tumor environment – the relative ratio of these cells is correlated to tumor grade. (5) Natural killer (NK) cells promote pro-inflammatory, anti-tumor GAM activation.

Gliomas are the most common form of brain tumor, with glioblastoma multiforme (GBM) – grade IV astrocytoma – presenting as the most malignant and fatal manifestation (208). The tumor microenvironment (TME) in GBM plays a vital role in GBM malignancy and remains a significant barrier to treatment. Notably, this microenvironment consists of a heterogeneous population of immune cells, including microglia, macrophages, MDSCs, CD4+ T cells, CD8+ T cells, Tregs, NK cells, and dendritic cells, suggesting a strong immunological component of GBM, as seen in other cancers (208) (**Figure 4**).

Of particular note, patient biopsies have revealed up to 30-50% of GBM tumor mass consists of glioma-associated microglia and macrophages (GAMs) (209), with higher numbers correlated to higher grade gliomas (210). Microglia, especially, are found in high numbers (30-40%) (209) and have been shown to adopt primarily M2 phenotypes, contributing to anti-inflammation/immunosuppression, and thus facilitating tumor growth (211). Tumor cells also appear to stimulate microglia mobility *via* upregulation of genes associated with migration and invasion capability (209). Additional immunosuppressive factors secreted by GAMs include IL-10, MMPs, and arginase-1 (ARG-1). Moreover, tumor cells and GAMs secrete chemokines (e.g., monocyte chemotactic protein-1; CCL2) capable of attracting MDSCs – immature macrophages, granulocytes, dendritic cells, and myeloid progenitors (212) – to the tumor site, where they can further promote tumor growth *via* release of anti-inflammatory cytokines (e.g., TGF-β, IL-10) (209). Disrupting this signaling from GAMs/microglia to other immune cells has received focus for therapeutic development (213, 214).

Infiltration of T cells into the tumor microenvironment also play a role in glioma outcomes. Particularly, the ratio of CD4+ to CD8+ T cells is highly correlated to glioma grading, with higher grade gliomas (i.e., GBM) having large numbers of CD4+ T cells (>93%) compared to CD8+ T cells in the TME (211, 215). In accordance with this finding, patient survival was observed to improve with higher CD8+ T cell counts, reflecting the potential of CD8+ T cells to attack tumor cells. These T cell population and activation states have been primarily attributed to the relatively high concentrations of anti-inflammatory cytokines (i.e., TGF-β, IL-10) secreted by tumor cells as well as GAMs, as described above (216). Tregs have also been found in the TME, though interestingly, their impact on prognosis has been debated. Several studies have corroborated the presence of Tregs in the TME and hypothesized that their immunosuppressive functions may contribute to glioma severity, which is supported by one study that found significantly higher Treg counts in GBM compared to lower grade gliomas (215); however, this and another study (216) did not conclude a difference in prognosis based on Treg counts, while a separate study did (217). Based on these differences, more research is needed to determine the importance of Tregs in GBM.

NK cells are also found in the tumor microenvironment and could potentially be able to attack and kill tumor cells. Indeed, glioma cells express natural killer group 2 member D (NKG2D) ligands, which bind to NKG2D receptors found on NK cells to initiate cytotoxic programs (i.e., pro-inflammatory cytokine and granule secretion (218). Despite this potential, high glioma expression of MHC class I molecules protects tumor cells from NK cytotoxicity (219). Genetic approaches such as overexpressing NKG2D ligands in tumor cells have been proposed to enhance endogenous NK cytotoxic functions to treat GBM (218). More recently, exogenous NK cell and antibody treatment has shown therapeutic potential (220). Most notably, perhaps, these results appear to be in large part due to crosstalk between NK cells and glioma-associated microglia and macrophages, converting the typical anti-inflammatory activation of these cells in the TME to a more pro-inflammatory activation capable of attacking GBM cells. This suggests that therapeutic approaches affecting immune crosstalk and multiple targets may be more successful than single-target approaches.

## Discussion

Once thought to be immune-privileged, it is now commonly known that the CNS facilitates both innate and adaptive immune responses involving a wide variety of effector cells. More importantly, neuroinflammation continues to be recognized as an important contributor to many major pathological conditions, including stroke and traumatic brain injury, neurodegenerative diseases, and cancer. Given this important role for neuroinflammation, neuroimmune-targeted therapies are receiving significant interest to treat these conditions. Despite strong potential and some success in pre-clinical and early clinical trials in conditions including Alzheimer’s disease and stroke (221, 222), many similar trials have failed or are only in early stages, with a long way to go to determine therapeutic efficacy.

One potential explanation for the relative lack of successful therapeutics is the focus on neuroinflammation and CNS-resident cells and mechanisms. While important, there is now compelling evidence that the peripheral immune system plays a strong role in all of these conditions, as well, and successful therapeutics may need to target the interactions between the central and peripheral immune systems. As an example, a recent study demonstrated a requirement for monocyte infiltration to induce neurodegeneration in a model of Parkinson’s disease (223), suggesting targeting the recruitment of monocytes and peripheral immune cells may be more effective than microglia and traditional CNS targets. Also of note in Parkinson’s patients is gastrointestinal dysfunction, leading to a revelation that the gut–brain axis contributes to pathology both *via* direct alpha-synuclein trafficking, as well as interactions with gut microbiota (224). Importantly, gut microbiota is now known to affect both the central and peripheral immune systems, and is being increasingly linked to neuroinflammation as well as neurological and neurodegenerative conditions – highlighting an additional avenue for therapeutic development (225).

While many of these interactions are becoming better understood, their complexity poses additional considerations to develop effective therapeutics. A major consideration is the cause or effect or “chicken and egg” issue – simply, it is often unclear whether these immune processes are a cause of pathology or result of underlying pathology, and in many cases it appears to be both (226). This makes it difficult to determine key targets and introduces timing considerations. Better understanding of the order and timing of these interactions will better inform treatment strategies. Finally, multi-faceted approaches are likely needed for most of these conditions, which have myriad contributing factors, etiologies, and symptoms. Simple enhancement or blunting of inflammatory processes with traditional drugs (e.g., minocycline) are unlikely to be effective due to the complex interplay of immune cells and processes, calling for more specific, targeted therapies. For example, stimulating M2 activation of microglia while targeting neutrophil recruitment in stroke may serve to enhance reparative processes and protect the blood-brain barrier, leading to improved recovery rates. Alternatively, activating pro-inflammatory and cytotoxic programs in microglia and macrophages would likely be more beneficial for GBM treatment and could perhaps be achieved *via* modifying infiltrating NK cells – taking advantage of crosstalk mechanisms where traditional treatment has failed or been difficult to implement. Ultimately, similar strategies targeting immune crosstalk offer significant potential and improvement to treat neurological and neurodegenerative disorders for which effective therapeutics have mostly remained elusive.

## Author Contributions

AP, AL, YY, and SS designed, wrote, and edited the manuscript and have approved it for publication. All authors contributed to the article and approved the submitted version.

## Funding

This work was funded by the National Science and Technology Center for Emergent Behaviors of Integrated Cellular Systems, Grant No. 0939511.

## Conflict of Interest

The authors declare that the research was conducted in the absence of any commercial or financial relationships that could be construed as a potential conflict of interest.
